# A preliminary deep learning study on automatic segmentation of contrast-enhanced bolus in videofluorography of swallowing

**DOI:** 10.1038/s41598-022-21530-8

**Published:** 2022-11-05

**Authors:** Yoshiko Ariji, Masakazu Gotoh, Motoki Fukuda, Satoshi Watanabe, Toru Nagao, Akitoshi Katsumata, Eiichiro Ariji

**Affiliations:** 1 Department of Oral and Maxillofacial Radiology, Aichi-Gakuin University School of Dentistry, 2-11 Suemori-dori, Chikusa-ku, Nagoya, 464-8651 Japan; 2 Department of Oral Radiology, School of Dentistry, Osaka Dental University, Osaka, Japan; 3 Department of Maxillofacial Surgery, Aichi-Gakuin University School of Dentistry, Nagoya, Japan; 4 Department of Oral Radiology, Asahi University School of Dentistry, Mizuho, Japan

**Keywords:** Health care, Medical research

## Abstract

Although videofluorography (VFG) is an effective tool for evaluating swallowing functions, its accurate evaluation requires considerable time and effort. This study aimed to create a deep learning model for automated bolus segmentation on VFG images of patients with healthy swallowing and dysphagia using the artificial intelligence deep learning segmentation method, and to assess the performance of the method. VFG images of 72 swallowing of 12 patients were continuously converted into 15 static images per second. In total, 3910 images were arbitrarily assigned to the training, validation, test 1, and test 2 datasets. In the training and validation datasets, images of colored bolus areas were prepared, along with original images. Using a U-Net neural network, a trained model was created after 500 epochs of training. The test datasets were applied to the trained model, and the performances of automatic segmentation (Jaccard index, Sørensen–Dice coefficient, and sensitivity) were calculated. All performance values for the segmentation of the test 1 and 2 datasets were high, exceeding 0.9. Using an artificial intelligence deep learning segmentation method, we automatically segmented the bolus areas on VFG images; our method exhibited high performance. This model also allowed assessment of aspiration and laryngeal invasion.

## Introduction

Dysphagia is a frequently observed clinical sign in patients with stroke, head-neck cancer, and various other medical conditions^1^. Dysphagia is defined as an impairment in swallowing function during eating and drinking, which causes subjective discomfort or objective difficulty in the formation or transportation of a bolus from the oral or pharyngeal cavities to the upper esophagus^1,2^. These errant events during swallowing lead to the accumulation of pharyngeal residue. Thereafter, this accumulation may accidentally enter the respiratory tract (aspiration)^1,2^. Aspiration can cause airway obstruction and pneumonia, which are associated with increased mortality^1,2^. Rapid and accurate assessment of swallowing function may reduce associated health risks^1^.

Instrumental evaluation of swallowing has been widely studied for many years^3^. Videofluorography (VFG) is a type of real-time X-ray video, which is regarded as the gold standard for assessing the oral and pharyngeal dynamics of swallowing^1,2^. This approach allows clinicians to view and evaluate the structure and function of all stages of swallowing^1^.

The quantitative evaluation of swallowing function using VFG has been studied^2^. Studies thus far have involved the quantification of pharyngeal residue^3^, laryngeal elevation and hyoid bone displacement during swallowing^3^, movement of the posterior pharyngeal wall during swallowing^4^, pharyngeal transit time^3^, and pharyngeal swallowing reaction time^5^. Clinicians must analyze swallowing videos in a frame-by-frame manner^2^, which requires considerable time and effort.

In recent years, deep learning technology has made remarkable progress using the medical images of numerous databases and deep convolutional neural networks^1,2,6^. These studies have provided compelling results for disease detection, assessment, and diagnosis^2^. There have also been some reports regarding swallowing. Zhang et al.^1^ focused on displacement of the hyoid bone during swallowing; they reported that the hyoid bone could be automatically detected in VFG images by using deep learning. Mao et al.^6^ proposed another approach to tracking hyoid movement with neck sensor support. Caliskan et al.^2^ successfully performed automated bolus detection in VFG images using a deep neural network (Mask-R-CNN). They targeted 450 swallowing images of 30 patients, yielding a Jaccard index (JI) value of 0.71. We hypothesized that it might be possible to observe aspiration and pharyngeal residue in real time if the bolus area could be automatically segmented with high performance exceeding the JI of 0.9.

This study created a deep learning model for automated bolus segmentation in VFG images of patients with healthy swallowing and dysphagia using the neural network for semantic segmentation (U-Net), then investigated whether the model could automatically visualize aspiration and laryngeal invasion findings.

## Results

### Times required for training and inference processes

An interval of 15 h and 43 min was needed from 500-epoch training until the creation of a trained model. An interval of 42 s was needed from the application of test dataset 1 to the trained model until evaluation of the model; for test dataset 2, an interval of 1 min and 27 s was needed.

### Evaluation of trained model

**Figure 1:**
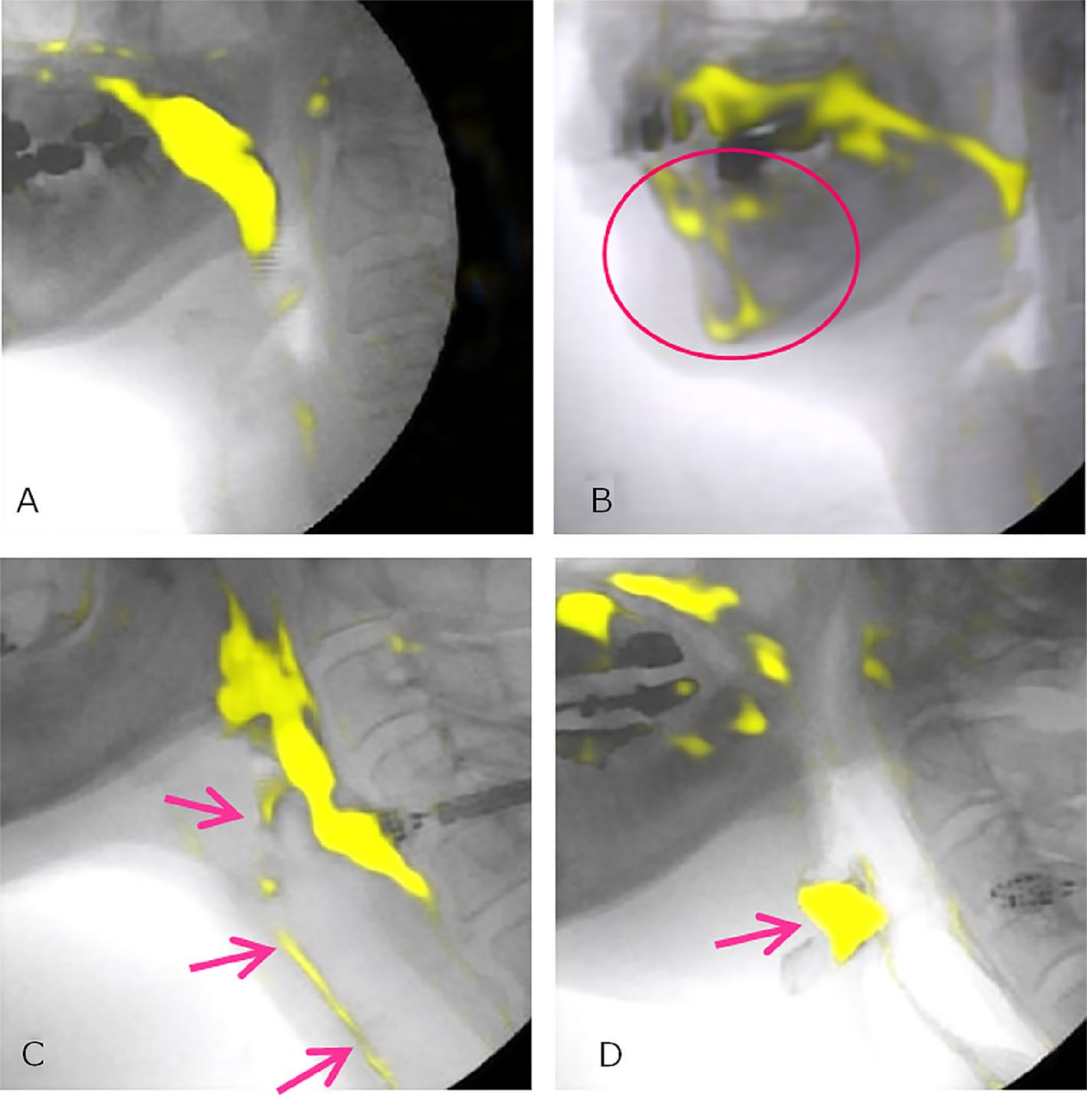
Examples of contrast bolus segmentation using deep learning (**A**) The contrast bolus was accurately predicted. (**B**) The contrast bolus was not accurately predicted. This may have been caused by false-positive detection of the metals and chin area. (**C**) The contrast bolus, including area showing aspiration swallowing, was accurately predicted. (**D**) The retention of contrast medium in the pyriform sinus was well-visualized.

Following the application of test dataset 1 of healthy swallowing to a trained model, the JI, DSC, and sensitivity were 0.90 ± 0.06, 0.94 ± 0.05, 0.95 ± 0.06, respectively (Table 1). Examples of contrast bolus segmentation using artificial intelligence deep learning are shown in Fig. 1. As demonstrated in Fig. 1A, the trained model was able to accurately predict the contrast bolus. In Fig. 1B, the trained model could not accurately predict the contrast bolus; this may have been caused by false-positive detection of the metals and chin area.

**Table 1:** Segmentation results.

Used test datasets	JI	DSC	Sensitivity
Test dataset 1	0.90 ± 0.06	0.94 ± 0.05	0.95 ± 0.06
Test dataset 2	0.92 ± 0.07	0.96 ± 0.03	0.95 ± 0.05

*JI* Jaccard index, *DSC* Sørensen–Dice coefficient.

Following the application of test dataset 2 of aspiration/laryngeal invasion to the trained model, the JI, DSC, and sensitivity were 0.92 ± 0.05, 0.96 ± 0.03, 0.95 ± 0.05, respectively (Table 1). As shown in Fig. 1C, the trained model was able to accurately predict the contrast bolus, including the area involved in aspiration swallowing. Figure 1D shows that the retention of contrast medium in the pyriform sinus was well-visualized.

## Discussion

In this study, we proposed a model for automatic bolus segmentation on swallowing VFG using a U-Net neural network for semantic segmentation^7,8^. The performance of the model was greater than 0.9. In addition, aspiration, and residual contrast medium in the piriform sinus were well-visualized. The development of a quantitative and qualified computer-assisted system can help clinicians to efficiently and rapidly assess swallowing videos in a busy clinical setting^1,2^. An integrated algorithm for VFG diagnosis will be feasible in the near future^5^.

Our model can clearly segment and visualize a bolus when there are no structures with similar density in VFG images^3^. Falsely segmented structures included the mandible, cartilage, and metals for tooth restoration (Fig. 1B)^2^. A contrast along the posterior wall of the pharynx without bolus formation was occasionally visible.

Various deep learning convolutional neural networks (CNNs) on swallowing have been reported. There are three main approaches. The first is reports that identifies the pharyngeal phase from swallowing records, including reports by Lee et al.^5^ and Bandini et al.^9^. The second is reports that detects the movement of the hyoid bone on VFG images, including report by Zhang et al.^1^. The third is reports that segments the bolus contour during swallowing, including report by Caliskan et al.^2^ and this study.

Lee et al.^5^ predicted the delay in response time of pharyngeal swallowing reflex using Inception-V1 (Google Net) CNN for classification. Bandini et al.^9^ compared the accuracy in predicting pharyngeal phase using various original CNNs architectures and input frames. As a result, it was reported that 2D-CNN using 3 frames as input had the highest accuracy.

Zhang et al.^1^ detected the hyoid bone using three types of object detection CNNs: that is, faster region based convolutional neural networks (Faster-RCNN), you look only onse (YOLO), and the single shot multibox detector (SSD). As a result, use of SSD produced highest performance with the mean average precision of 89.1. However, object detection CNNs seems unsuitable for tracking contrast-enhanced bolus because the results are displayed in squares.

This study used a U-Net neural network for semantic segmentation, which provides the area of each class on a pixel-by-pixel basis without the use of bounding boxes^7,8^. Multiple types of objects are segmented but categorized in a single class. A U-Net categorizes objects based on information regarding pixels and surrounding pixels, then finds the object areas by performing continuous local processing^7,8^. Because the target area of this study was limited to the bolus of contrast medium, there was no requirement for consideration of multiple classes.

Caliskan et al.^2^ segmented boluses using Mask R-CNN, a neural network for instance segmentation. Instance segmentation identifies each class area in a pixel-by-pixel manner and distinguishes different objects. Mask R-CNN provides an object area in a two-stage configuration by creating a bounding box and performing segmentation for each detection area. Our study revealed higher values than the values reported by Caliskan et al.^2^.

Regarding the difference in CNNs of segmentation, Kromp et al.^10^ compared the clinical segmentation performance in nuclear medicine images using multiple neural networks, including U-Nets and Mask R-CNNs. They found that U-Nets achieved higher mean Dice scores, whereas the neural networks for instance segmentation were better in complex images. Further analyses are expected to use instance segmentation neural networks, including Mask R-CNN, depending on the targets.

The difference in frame rates has not yet been clarified. In our study, the frame rate is low (15 frames/sec; FPS), but in other reports it is 30 FPS^1,2^. In the future, use of 30FPS or ideally 60FPS data will further improve performance and expect clinical application.

Interobserver variability in qualitative assessment of swallowing function on VFG images has been reported^1,5^. Therefore, in this study, the presence or absence of dysphagia was decided after discussion. The results of segmentation are expected to fluctuate depending on the annotation quality. In this study, one radiologist artificially colored the bolus area, while another radiologist confirmed them. The discrepancy at annotation was less than 0.5% of the total images.

This study investigated the VFG images at command swallowing of barium liquid. There may be the discrepancy in results between normal feeding and command swallowing. To minimize this discrepancy, the examination of spontaneously chewing and swallowing using semi-solid material kneaded with barium should be added.

The distribution of patient dysfunction varies among studies, which can influence the results. The small number of patients thus comprised a limitation in this study. Furthermore, this study only analyzed data from a single institution. Multicenter studies are needed to confirm our findings. Differences among VFG devices may lead to variations in image quality or inconsistency^1,2^. Furthermore, image quality depends on each clinician’s ability to control the radiation dose administered to patients^1^. It is also difficult to standardize patient position among studies^4^. Although our institution’s protocol involves sitting in a chair and swallowing to help maintain a consistent position, there were some static movements in patients who were unable to swallow well. Bolus segmentation was achieved in this study, but it may be necessary to introduce a time axis for assessment of overall swallowing function.

In conclusion, this study generated a promising deep learning model for semantic segmentation of bolus on VFG images to assess swallowing function. This study has three potentials that will contribute to clinical practice in the future. First, the system of this study provides color images segmented the bolus areas from raw VFG images. Second, the results will assist the diagnosis of clinicians, especially those unfamiliar with VFG imaging, by observing the movement of the bolus areas and anatomical structures. Third, they may be useful in determining rehabilitation strategies for patients with dysphagia or in post-treatment evaluation.

## Methods

### Ethical considerations

This study was conducted with approval of Ethics Committee of Aichi Gakuin University School of Dentistry, (No 586) and in accordance with the Declaration of Helsinki. This study is a non-invasive observational study using only existing anonymized video data. By using opt-out, subjects were given the opportunity to refuse to participate in the study. The Ethics committee of Aichi Gakuin University School of Dentistry has waived the requirement for the informed consent from all participants.

### Participants

The participants were 12 patients (seven men and five women; mean age, 58.4 ± 23.3 years; age range, 20–89 years) who visited the swallowing outpatient clinic at our hospital between November 2018 and January 2020; all underwent videofluorography (VFG) for examination of swallowing function.

### Videofluorography

The patients sat on a chair for VFG (MK-102, Tomomi-koubou, Shimane, Japan) in a normal eating position without head fixation; they were examined with a fluorographic machine (DCW-30A, Canon Medical Systems, Tokyo, Japan).

The contrast sample was made with 50 mL of 50% w/v barium sulfate (Baritogen Deluxe, Fushimi Laboratory, Kagawa, Japan) mixed with thickener (Throsoft Liquid 12 g/pack, Kissei Pharmaceutical Co. Ltd, Nagano, Japan). The concentration of this barium is much thinner than that (200w/v% − 240w/v%) usually used for the upper gastrointestinal tract. This might contribute to avoid the adhesion of barium to the mucosa of the oral and pharyngeal cavities and to provide sufficiently qualified images. The examiner placed a spoonful of sample (approximately 5 mL) into the patient’s mouth, and the patient began to swallow it at the examiner's signal. The swallowing examinations with this sample were performed three times, and moving images were recorded.

Subsequently, examinations were performed using a 50-mL sample of 50% w/v barium sulfate (Baritogen Deluxe). The patient was instructed to put a sample of a paper-cup (about 5 mL) in into his/her mouth, and began to swallow it at the examiner's signal. The swallowing examinations with this sample were performed three times, and moving images were recorded. Consequently, six swallowing examinations were performed for each patient.

Diagnoses of swallowing function based on VFG images were made by the mutual consent of two radiologists and one oral surgeon with more than 20 years of experience. The presence or absence of residual contrast-enhanced bolus and aspiration/penetration events were assessed on VFG images. The severity of dysphagia was based on the penetration-aspiration scale^11^: seven patients in this study had healthy swallowing function, while 5 patients showed aspiration or laryngeal invasion.

### Image preparation

**Figure 2:**
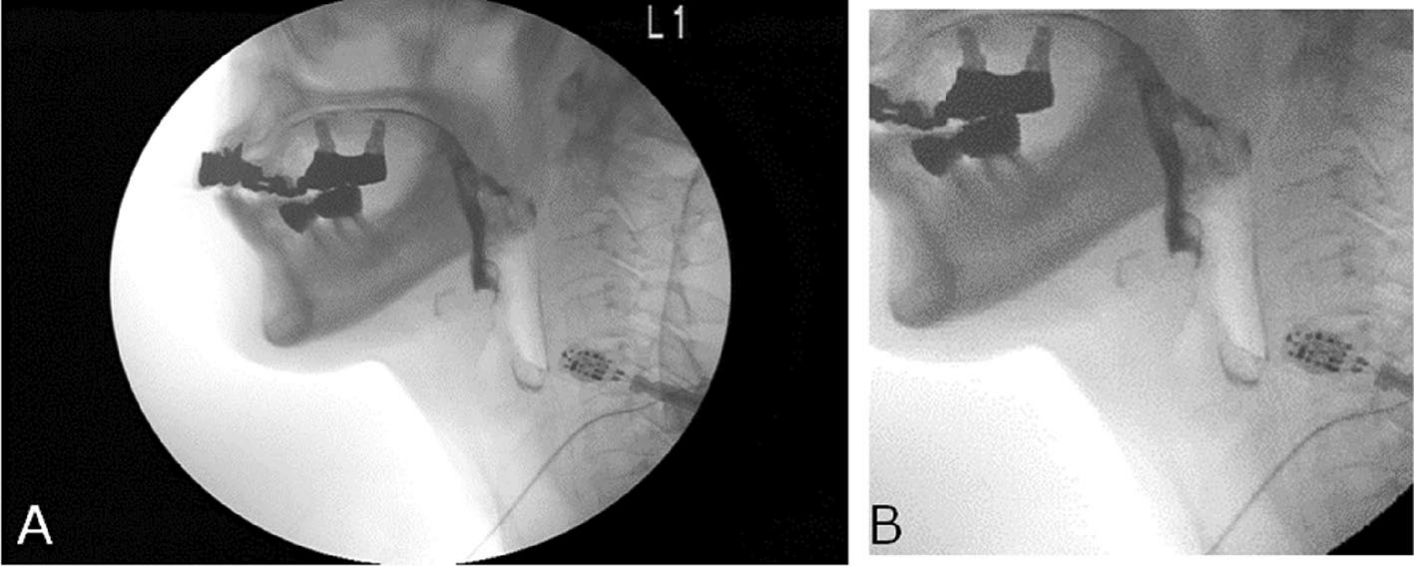
Image preparation. (**A**) A static image converted from videofluorography video images. The image size is 720 × 480 pixels. (**B**) The image was standardized to a size of 256 × 256 pixels by cutting off extra space at the top and front of the original image, then converted to JPEG format.

The VFG images (oral to pharyngeal phases) were continuously converted into 15 static images per second. The static images were standardized to a size of 256 × 256 pixels by cutting off extra space at the top and front of the images, then saved in JPEG format (Fig. 2).

### Allocation to training, validation, and test datasets

Images were arbitrarily assigned to training, validation, and test datasets (Table 2). For the training dataset, 1845 static images were used, including 1005 static images of 18 swallows in three patients with healthy swallowing, and 840 static images of 12 swallows in two patients with aspiration or laryngeal invasion. For the validation dataset, 155 static images of six swallows in one patient with healthy swallowing were used. As test dataset 1, 510 static images of 18 swallows in three patients with healthy swallowing were used. As test dataset 2, 1400 static images of 18 swallows in three patients with aspiration or laryngeal invasion were used.

**Table 2:** Allocation to training, validation, and test datasets.

	No. of patients	No of swallowing	No. of still images
Training dataset	3 patients with healthy swallowing	18	1005
	2 patients with aspiration/delayed swallowing	12	840
Total	5 patients	30	1845
Validation dataset	1 patient with healthy swallowing	6	155
Test dataset 1	3 patients with healthy swallowing	18	510
Test dataset 2	3 patients with aspiration/delayed swallowing	18	1400

### Deep learning system

**Figure 3:**
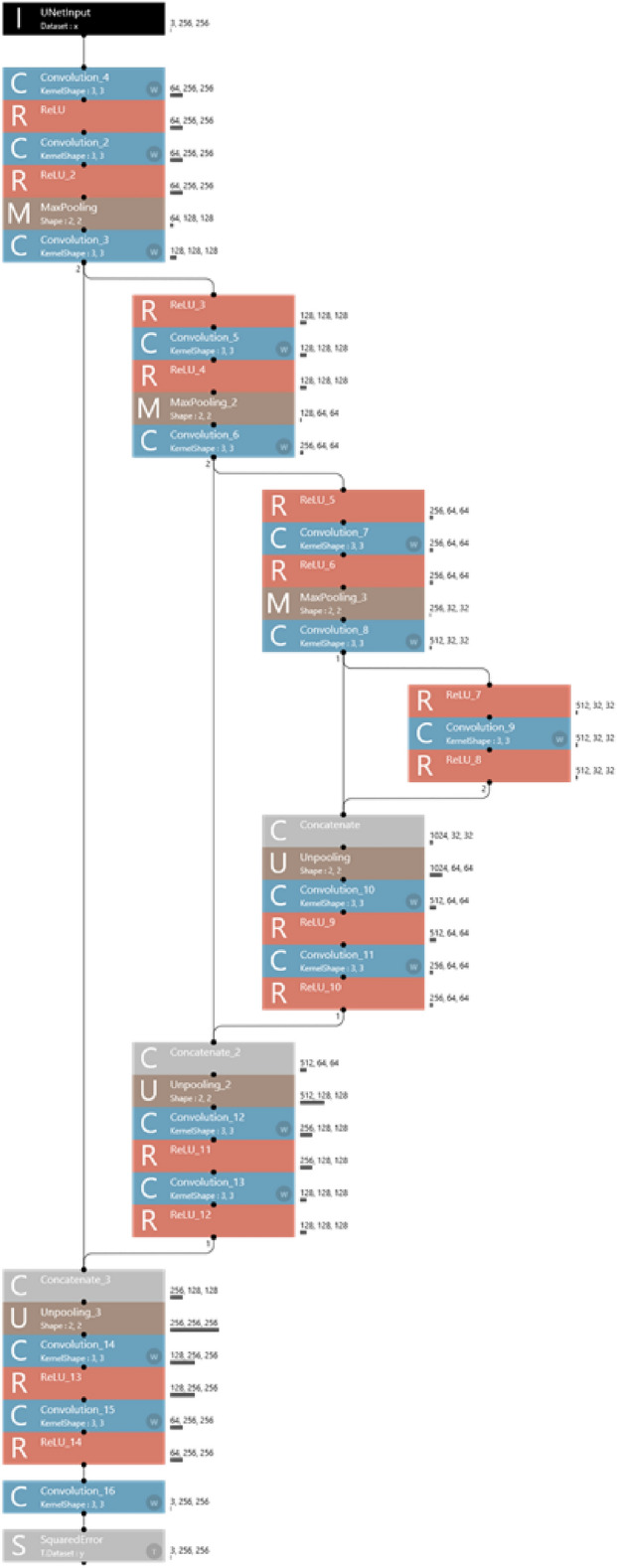
The U-Net neural network used for this study.

The deep learning system was built on a Windows PC with an 11 GB GPU of NVIDIA GeForce (NVIDIA, Santa Clara, CA, USA) and 128 GB of memory. The deep learning segmentation procedure was performed using a U-Net created on the neural network Console (Sony, Tokyo, Japan). U net is a neural network for fast and precise segmentation of images, and composed of encoder-decoder format symmetry structure, as shown in Fig. 3.

### Annotation

**Figure 4:**
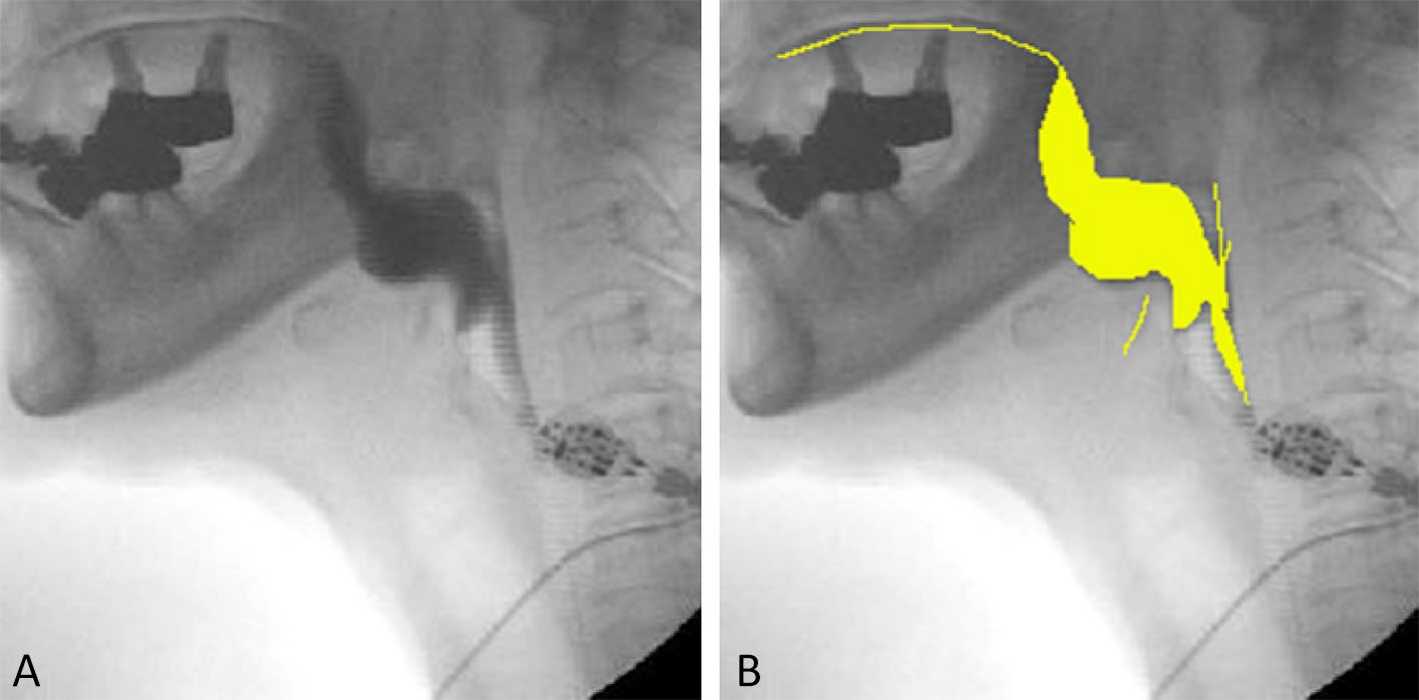
Annotation using training and verification datasets. (**A**) Original image. (**B**) Image in which contrast-enhanced bolus areas were colored.

For the training and verification datasets, images were created in which the contrast-enhanced bolus areas were segmented and colored using Photoshop (Adobe, Tokyo, Japan); these were used in addition to the original images (Fig. 4). In the annotation work, one radiologist with over 30 years of experience performed the segmentation of the contrast-enhanced bolus areas. Another radiologist with over 20 years of experience confirmed them. The bolus of the still images had very strong contrast and was easy to grasp. If the latter determined that the annotations were incorrect, the two radiologists discussed and corrected them. The number of revisions was less than 0.5% of the total images.

### Training process

**Figure 5:**
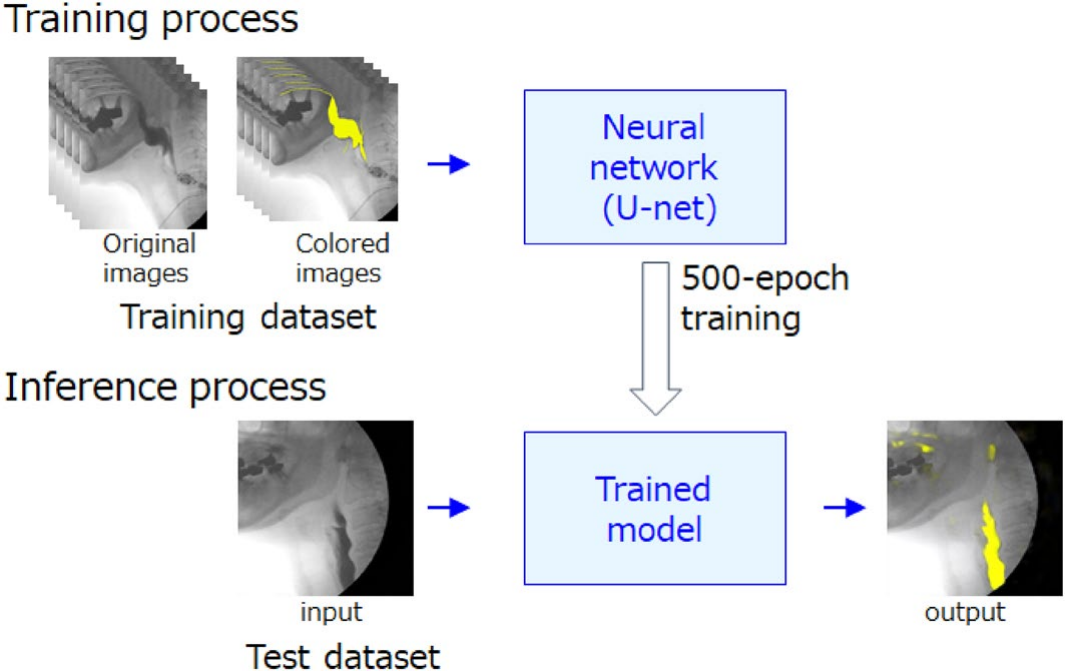
Training and inference processes. Following a 500-epoch training process using training and validation datasets paired with the original and colored images, a trained model was created. In the inference process, test dataset 1 or 2 was applied to the trained model and the model performance was evaluated.

The training process was performed with a U-Net neural network using training and validation datasets paired with the original and colored images (Fig. 5). U-net is a convolutional neural network for performing semantic segmentation of lesions or tissues on images, and has an almost symmetrical structure of the encoder-decoder module^7,8^. The encoder module progressively downsamples the image and reduces feature map resolution to capture high-level details of the image. The decoder module consists of a set of layers that upsamples the feature map of encoder to recover spatial information. Learning continued until the training loss was sufficiently small on the learning curve, and finally 500 epochs of learning were conducted. Thereafter, a trained model was created.

### Inference process

In the inference process, test dataset 1 or 2 was applied to the trained model to evaluate the model (Fig. 5). Prior to evaluation, the ground-truth of the contrast-enhanced bolus areas were identified on the test images by a radiologist. For evaluation of the model, Jaccard index (JI), Sørensen–Dice coefficient (DSC), and sensitivity were calculated according to the following equations^12^:

$$ {\text{JI}} = {\text{S}}\left( {{\text{P}} \cap {\text{G}}} \right)/{\text{S}}({\text{P}} \cup {\text{G}}) $$

$$  {\text{DSC}} 2 \times {\text{S}}\left( {{\text{P}} \cap {\text{G}}} \right)/\left( {{\text{S}}\left( {\text{P}} \right) + {\text{S}}\left( {\text{G}} \right)} \right) $$

$$ {\text{Sensitivity}}{\text{S}}\left( {{\text{P}} \cap {\text{G}}} \right)/{\text{S}}\left( {\text{G}} \right) $$

where S(P) was the colored bolus area on images predicted by the learning model, and S(G) was the ground-truth bolus area. S(P ∩ G) was the overlapped area of P and G, and S(P ∪ G) was the combined area. The ground-truth images and the predicted images by the deep learning model were superimposed, and the number of pixels in the above areas were calculated using Photoshop.

## Data Availability

The datasets generated and/or analyzed during the current study are not publicly available due to not permitted by the current ethical approval, but are available from the corresponding author on reasonable request.
